# Pitfalls and Pearls for Diagnosis of a Child with Tuberculosis Osteomyelitis Masquerading as Brodie’s Abscess: A Case Report

**DOI:** 10.5704/MOJ.2603.022

**Published:** 2026-03

**Authors:** SH Marseno, TW Martanto, A Zulkarnain, H Yazid, RY Christanto

**Affiliations:** 1 Department of Orthopaedics and Traumatology, Universitas Airlangga, Surabaya, Indonesia; 2 Department of Orthopaedics and Traumatology, Dr. Soetomo General Academic Hospital, Surabaya, Indonesia; 3 Department of Orthopaedics and Traumatology, Universitas Gadjah Mada Academic Hospital, Yogyakarta, Indonesia

**Keywords:** tuberculous osteomyelitis, Brodie’s abscess, paediatric bone infection, diaphyseal TB, PCR diagnosis

## Abstract

Tuberculous osteomyelitis is an uncommon manifestation of extrapulmonary tuberculosis in children, with diaphyseal involvement being particularly rare. Its insidious course and nonspecific imaging often lead to misdiagnosis as pyogenic osteomyelitis or Brodie’s abscess, resulting in delayed treatment. This report highlights a diagnostically challenging case of paediatric tibial tuberculosis osteomyelitis mimicking Brodie’s abscess. A 15-year-old girl presented with an 18-month history of chronic pain, swelling, and a draining sinus on her left lower leg. Despite undergoing multiple surgeries and a prolonged course of anti-tuberculous therapy, symptoms persisted. Radiographic and MRI findings suggested Brodie’s abscess with diaphyseal and metaphyseal involvement. However, biopsy revealed granulomatous inflammation, and polymerase chain reaction (PCR) testing confirmed Mycobacterium tuberculosis. Subsequent treatment led to clinical improvement and radiological bone union at six-month follow-up. The case underscores the diagnostic difficulty in distinguishing TB osteomyelitis from subacute pyogenic infections, especially in children. Classical radiologic features such as sclerotic rims, lytic lesions, and periosteal reaction are often indistinguishable. FNAB and pus cultures may yield inconclusive results. In this case, PCR was pivotal for definitive diagnosis. Misdiagnosis can lead to inappropriate treatment and unnecessary surgical interventions. Tuberculosis should be considered in the differential diagnosis of chronic osteomyelitis, particularly in TB-endemic regions. Early use of molecular diagnostics such as PCR, along with histopathology, is critical for accurate diagnosis. A multidisciplinary approach facilitates timely intervention, prevents complications, and improves outcomes.

## Introduction

Tuberculosis (TB) remains a major global health issue, with 10.6 million cases reported in 2021, an increase of 600,000 from 2020. Alarmingly, only 60.3% of these cases received treatment. Indonesia ranks second globally in TB burden, with cases increasing from 824,000 in 2020 to 969,000 in 2021. Extrapulmonary TB, including tuberculous osteomyelitis, is also rising. Its presentation often mimics other bone conditions, such as Brodie’s abscess, making diagnosis difficult. Tuberculous osteomyelitis primarily affects the metaphyses of long bones, while diaphyseal involvement remains rare.

This report presents a paediatric case initially misdiagnosed as Brodie’s abscess, later confirmed as TB osteomyelitis^1^.

## Case Report

A 15-year-old girl presented with chronic left lower leg pain, swelling, and a non-healing sinus that had persisted for 18 months. The symptoms initially began as mild pain without any history of trauma. She had received BCG vaccination, had no TB contacts, and did not exhibit systemic symptoms such as fever or weight loss. Family screening yielded no TB infection.

The patient underwent her first surgery in May 2022 to address bilateral ankle swelling; however, symptoms recurred shortly thereafter. A second procedure in July 2022 led to the diagnosis of bone TB, and anti-TB treatment (OAT) was initiated in August 2022. Despite treatment, swelling reappeared in July 2023, and fluid evacuation was repeated. After 18 months of therapy, persistent drainage from the calf sinus indicated ongoing inflammation. Joint motion remained restricted, particularly in the knee and ankle, causing significant difficulty with ambulation.

**Fig. 1:**
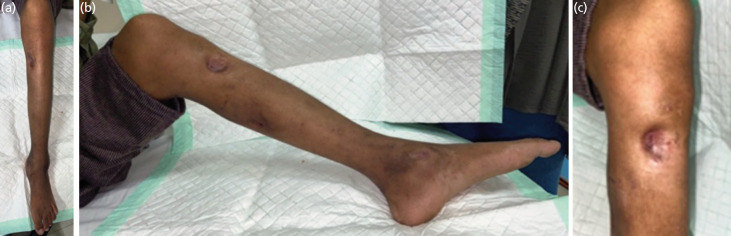
Clinical Appearance. (a) Anterior view of the left lower leg. (b and c) Medial view of the left lower shows a suppurative chronic lesion.

**Fig. 2:**
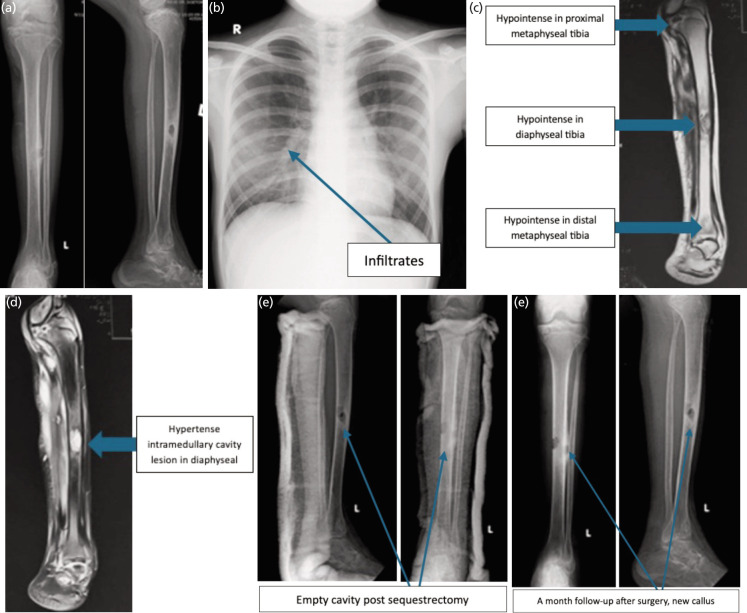
(a) Cruris, foot, and ankle before surgery, (b) Chest radiograph after 12 month of anti-TBC drug, (c) MRI T1-weighted image coronal view of tibia. The blue arrow shown hypointense in proximal and distal metaphyseal and diaphyseal of tibia. MRI T2 weighted image's coronal view of tibia. (d) The blue arrow showing hyperintense intramedullary cavity lesion. (e) empty cavity after sequestrectomy, (f) bone callus formed after a month of surgery.

On examination, a 2cm anteromedial tibial sinus with serous discharge was noted. Radiographs obtained in September 2023 revealed multiple lytic lesions with sclerotic margins involving the tibial metaphysis and diaphysis, cortical break, periosteal reaction, and lucent lesions in the distal tibia and fibula epiphyses. Notably, the joint spaces remained preserved (Fig. 1, Fig. 2a).

In July 2023, chest radiography revealed infiltrates in the right parahilar region, suggestive of possible pulmonary involvement and warranting further evaluation (Fig. 2b).

MRI of the left lower leg revealed extensive intraosseous abscesses with rim enhancement, suggestive of musculoskeletal TB. Interestingly, imaging characteristics mimicked Brodie’s abscess, defined by lytic lesions with cortical destruction and periosteal reaction, but the wider and more aggressive pattern pointed toward TB (Fig. 2c and 2d). Initial laboratory investigations, including CBC, liver and kidney function, and HIV serology, were within normal limits. FNAB revealed only chronic inflammation. PCR testing ultimately confirmed Mycobacterium tuberculosis. Initial differential diagnosis (April 2022) considered spindle cell tumour or giant cell tumour. However, granulomatous lymphadenitis (July 2022), granulomatous inflammation on biopsy, and subsequent positive molecular testing (gyrB gene) supported a diagnosis of TB.

Pus culture (Oct 2022) showed scanty acid-fast bacilli; rifampicin-sensitive M. tuberculosis was later detected in sputum (Aug 2023). An axillary lymph node biopsy (June 2023) showed granulomatous inflammation. Inflammatory markers (ESR, CRP) increased over time, while blood counts gradually declined. Despite these changes, biochemical parameters remained within normal limits.

**Fig. 3:**
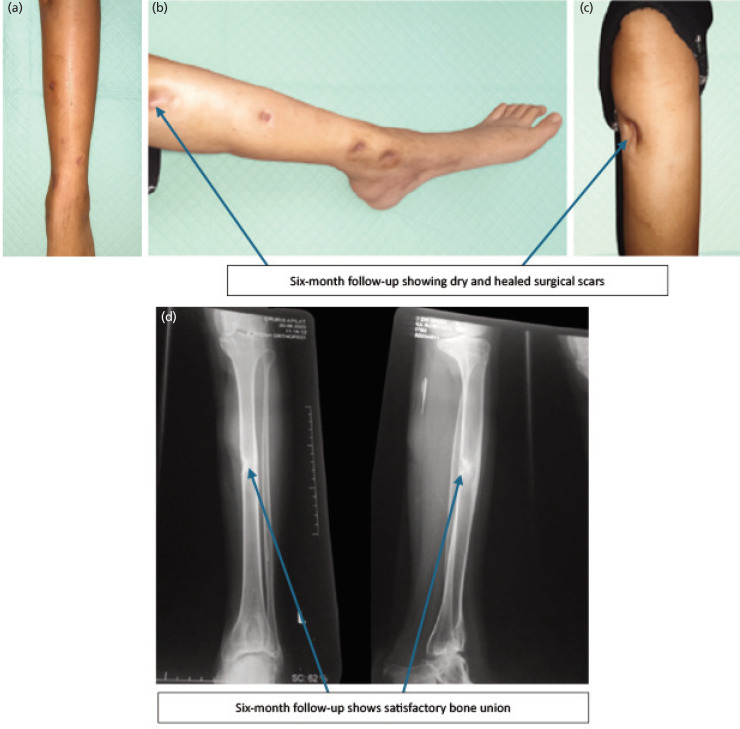
(a) Clinical and radiological appearance at six-month follow-up. Anterior view of the left lower leg showing well-healed surgical scars. (b and c) Medial and lateral views showing no signs of active infection and complete wound closure. (d) Anteroposterior and lateral radiographs demonstrating satisfactory bone union.

At the one-month follow-up, the surgical wound was found to be dry, and a plain radiograph of the tibia showed callus formation (Fig. 2e and 2f), indicating favourable clinical progress. At the six-month follow-up, the surgical scars were dry and well-healed without signs of infection (Fig. 3a, 3b, and 3c). Radiographs showed satisfactory bone union with continuous cortical bridging and no signs of complications (Fig. 3d and 3e).

## Discussion

Tuberculous osteomyelitis accounts for approximately 10% of extrapulmonary TB cases and usually spreads hematogenously from the lungs. In children, it typically affects the metaphysis while diaphyseal involvement remains rare. Due to its chronic and subtle nature, TB osteomyelitis is often misdiagnosed as pyogenic osteomyelitis or Brodie’s abscess, delaying appropriate treatment^1,2^.

In this case, the radiological findings, particularly MRI, closely mimicked Brodie’s abscess. Similar cases reported by Sari *et al* and Ajit *et al* emphasise that both conditions share overlapping imaging and clinical features. Zairi *et al* also noted that tuberculous osteomyelitis may resemble malignancies, making histopathological and molecular testing critical for accurate diagnosis^2-4^.

The patient’s clinical presentation, including localised swelling, chronic sinus drainage, and absence of systemic TB signs, illustrates the diagnostic challenges. Misleading FNAB findings and non-specific imaging added to the complexity of the diagnostic process. Histopathological analysis alone proved insufficient; definitive confirmation was achieved only through polymerase chain reaction (PCR) testing. This observation aligns with literature suggesting that definitive diagnosis requires molecular confirmation, particularly in atypical or chronic cases^2-4^.

According to Rasool’s classification, the radiologic features in this patient align with Type 1B Brodie’s abscess. While such imaging suggests subacute osteomyelitis, it does not rule out TB, especially in endemic regions. The overlap underscores the need for high clinical suspicion and comprehensive evaluation using imaging, microbiology, and PCR ^2-4^.

Standard TB osteomyelitis management involves 9–12 months of OAT. This patient received 18 months of therapy, reflecting both delayed diagnosis and recurring disease activity. Surgical interventions were performed before and during treatment, highlighting the importance of early identification to avoid unnecessary procedures and optimise treatment outcomes ^2-4^.

Previous studies by Vohra *et al* confirm that distinguishing TB osteomyelitis from Brodie’s abscess using imaging alone is difficult. A multidisciplinary approach is essential, integrating clinical, radiological, pathological, and molecular data to avoid diagnostic delays and manage complications effectively^1^.

In conclusion, this case highlights the diagnostic complexity of tuberculous osteomyelitis mimicking Brodie’s abscess. The patient’s prolonged course, delayed diagnosis, and persistent symptoms underscore the necessity of considering TB in atypical osteomyelitis cases, especially in endemic areas. PCR and biopsy remain essential for diagnosis. Early and accurate identification not only prevents unnecessary surgical interventions but also ensures appropriate treatment duration and improves clinical outcomes. A coordinated, multidisciplinary strategy is essential for timely diagnosis and effective management in such complex cases.
